# Supplementary material for: Virological characteristics of SARS-CoV-2 Omicron BA.5.2.48
Source: Front Immunol. 2024 Oct 1;15:1427284. doi: 10.3389/fimmu.2024.1427284 (PMC11473351; doi:10.3389/fimmu.2024.1427284)
Supplement: Supplementary file 1 [file DataSheet1.pdf]

Supporting Information for

Virological characteristics of SARS-CoV-2 Omicron BA.5.2.48

Wenqi Wang[1, 2, a], Qiushi Jin[2, 3, a], Ruixue Liu[2, 4, a], Wentao Zeng[5, a], Pengfei Zhu[2, 4], Tingting Li[2, 5], Tiecheng Wang[2], Haiyang Xiang[2], Hang Zhang[6], Qin Chen[5], Yun Gao[5], Yana Lai[5], Fang Yan[4], Xianzhu Xia[2, 3], Jianmin Li[5, *], Xuefeng Wang[2, *], Yuwei Gao[2, 4, *]

1. College of life sciences, Northeast Normal University, Changchun, China
2. Changchun Veterinary Research Institute, Chinese Academy of Agricultural Sciences, Changchun, China
3. College of Veterinary Medicine, Northeast Agricultural University, Harbin, China
4. College of Veterinary Medicine, Shanxi Agricultural University, Jinzhong, China
5. State Key Laboratory of Reproductive Medicine and Offspring Health, Jiangsu Laboratory Animal Center, Jiangsu Animal Experimental Center of Medicine and Pharmacy, Department of Cell Biology, Animal Core facility, Key Laboratory of Model Animal, Collaborative Innovation Center for Cardiovascular Disease Translational Medicine, National Vaccine Innovation Platform, Nanjing Medical University, Nanjing, China
6. Senior Cadre Department, The 964(th) Hospital of Joint Logistics Support, PLA, Changchun, China

[a]These authors contributed equally to the work.

Correspondence: yuwei0901@outlook.com (YG), xuefeng_wangNUDT@outlook.com (XW) and Jianminli@njmu.edu.cn (JL)

This PDF file includes:

Figs. S1 to S6
Tables S1 to S3

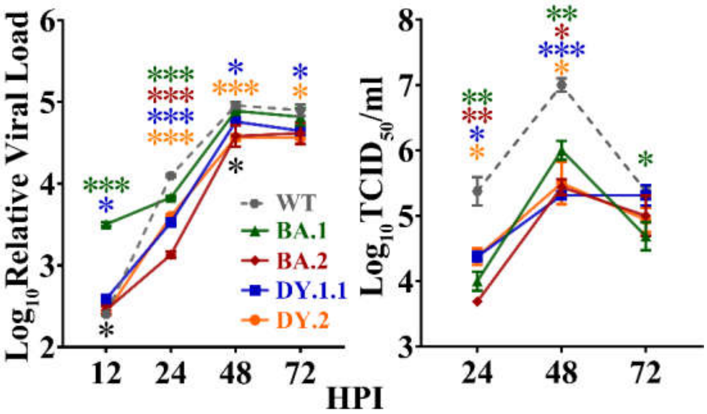

Fig. S1. Replicative kinetics of WT and Omicrons in Vero E6 cells. Significances of replicative differences between WT (gray) and Omicrons are revealed. See Fig. 1 for more details.

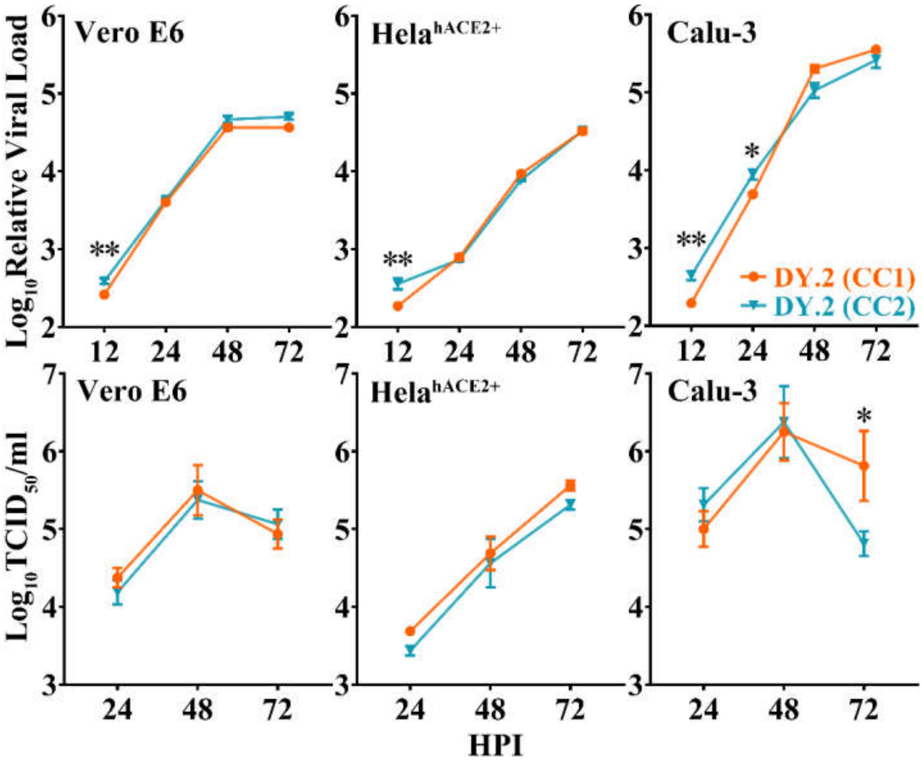

Fig. S2. Replicative kinetics of two DY.2 isolates in three kinds of cell lines. Significances of replicative differences between DY.2-CC1 (orange) and DY.2-CC2 (cyan) are revealed.

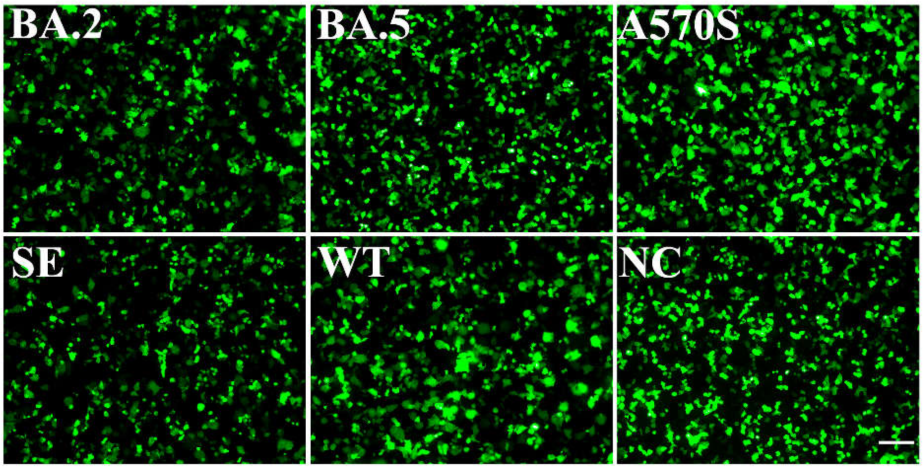

Fig. S3. Spike mediated cell-cell fusion demonstrated on spike-expressed Hela^{hACE2+} cells. BA.2, BA.5, BA.5-A570S (A570S), BA.5-A570S-K147E (SE), D614G spikes and a negative control (NC, without glycoproteins) are included. Scale bar: 200 μm.

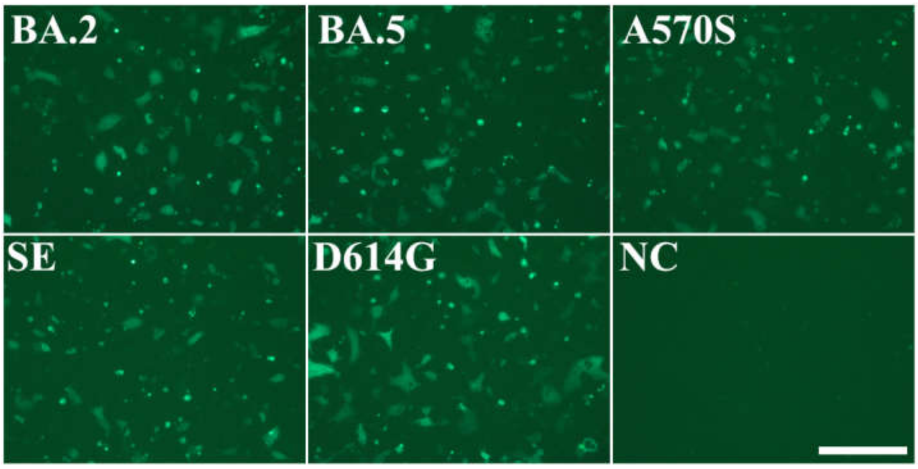

Fig. S4. Spike mediated cell-cell fusion based on the DSP method. BA.2, BA.5, BA.5-A570S (A570S), BA.5-A570S-K147E (SE), D614G spikes and a negative control (NC) are included. Scale bar: 100 μm.

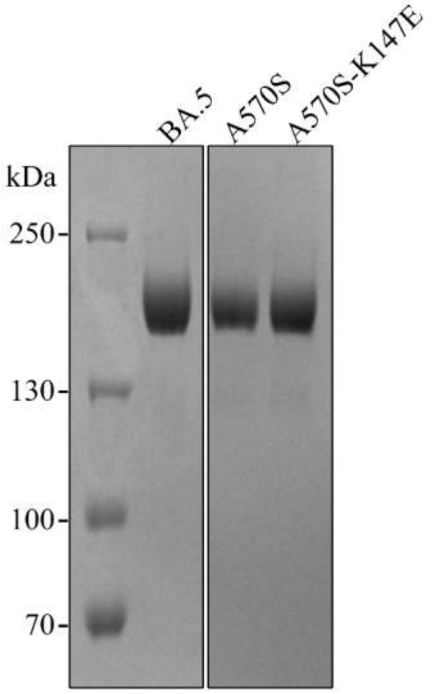

Fig. S5. Purification of BA.5, BA.5-A570S (A570S) and BA.5-A570S-K147E (A570S-K147E).

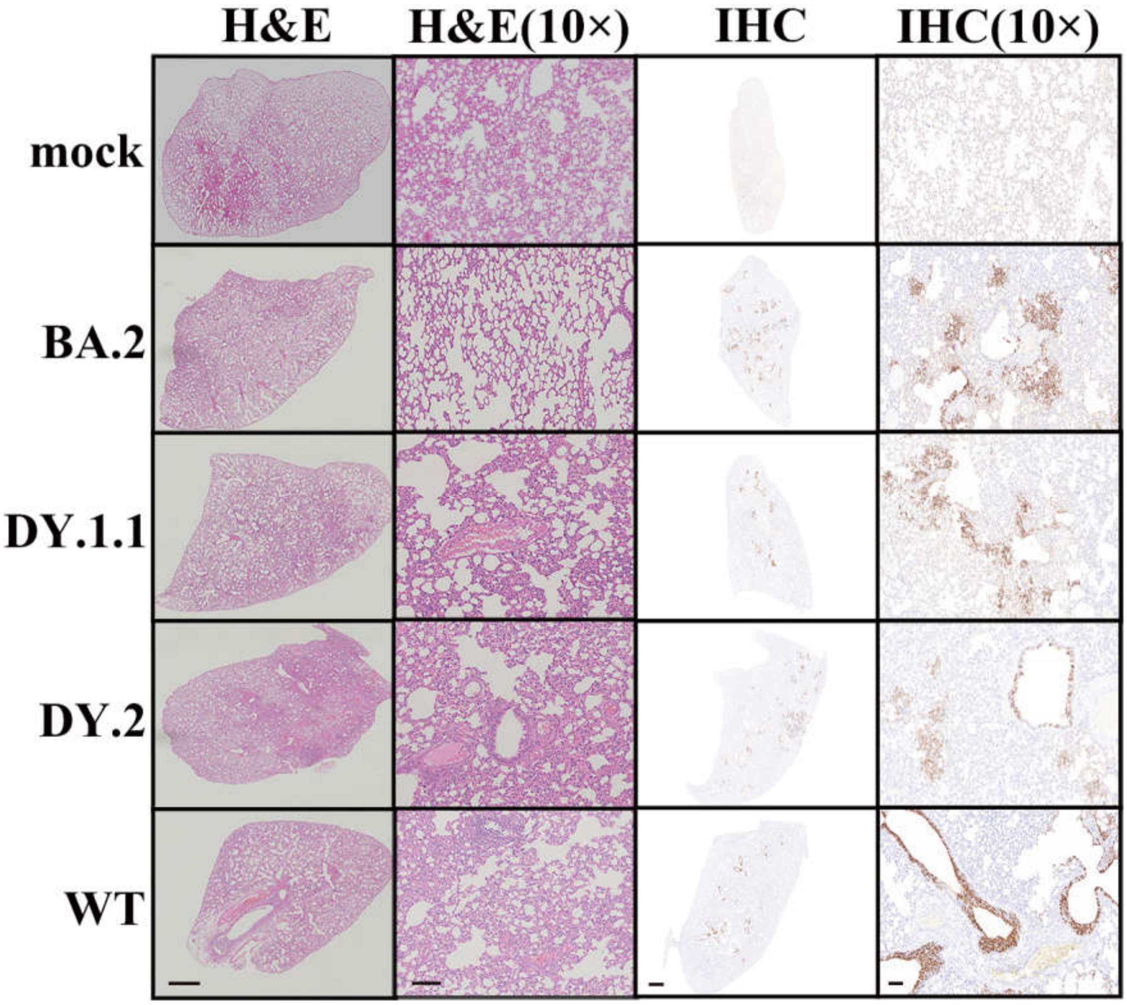

Fig. S6. H&E staining and immunohistochemistry (IHC) of the lungs of infected K18-hACE2 hamsters. Scale bars: 1 mm (panoramic images) and 100 μm (10× enlarged images).

Table S1. Primer applications and sequences.

Primer applications	Primer sequences (5' to 3')
site-directed mutagenesis to construct KA570S	gcagtttggccgggacattagcgacaccacc
	ggtggtgtcgctaatgtcccggccaaactgc
site-directed mutagenesis to construct K147E	tggacgtgtactaccacgagaacaacaagtcttgg
	ccaagacttgttgttctcgtggtagtacacgtcca
RT-PCR to amplify spike fragment flanking $spike_{570}$	CTTCTACATGCACCAGCAACTG
	ACTTCTGTGCAGTTAACACCC

Table S2. Antibodies information

Name	Vendor	Application
anti-nucleocapsid antibody	Genetex	immunohistochemistry, immunofluorescence and immunoblot
anti-spike S2 antibody	Genetex	immunoblot
HRP-conjugated goat-anti-rabbit antibody	ZSGB-Bio	immunohistochemistry
FITC-conjugated goat-anti-rabbit antibody	Jackson ImmunoResearch	immunofluorescence

Table S3. Expression of ACE2 and TMPRSS2 genes in three cell lines (in FPKM).

	Vero E6	$Hela^{hACE2}$	Calu-3
ACE2	0.31	96	5.08
TMPRSS2	0	0	6.62
